# Multiple Early Introductions of SARS-CoV-2 to Cape Town, South Africa

**DOI:** 10.3390/v13030526

**Published:** 2021-03-22

**Authors:** Susan Engelbrecht, Kayla Delaney, Bronwyn Kleinhans, Eduan Wilkinson, Houriiyah Tegally, Tania Stander, Gert van Zyl, Wolfgang Preiser, Tulio de Oliveira

**Affiliations:** 1 Division of Medical Virology, Faculty of Medicine and Health Sciences, Stellenbosch University, Cape Town 8000, South Africa; kayladelaney860@gmail.com (K.D.); 16478517@sun.ac.za (B.K.); guvz@sun.ac.za (G.v.Z.); preiser@sun.ac.za (W.P.); 2 Tygerberg Business Unit, National Health Laboratory Service (NHLS), Cape Town 8000, South Africa; Tania.Stander@nhls.ac.za; 3 KwaZulu-Natal Research Innovation and Sequencing Platform (KRISP), School of Laboratory Medicine and Medical Sciences, University of KwaZulu-Natal, Durban 4000, South Africa; ewilkinson83@gmail.com (E.W.); houriiyah.tegally@gmail.com (H.T.); 4 Centre for Aids Programme of Research in South Africa (CAPRISA), Durban 4000, South Africa; 5 Department of Global Health, University of Washington, Seattle, WA 98195, USA

**Keywords:** betacoronavirus, SARS-CoV-2, COVID-19, genome sequencing, mutation, phylogenetics, Cape Town, Western Cape Province, South Africa, molecular epidemiology

## Abstract

Cape Town was the first city in South Africa to experience the full impact of the coronavirus disease 2019 (COVID-19) pandemic. We acquired samples from all suspected cases and their contacts during the first month of the pandemic from Tygerberg Hospital. Nanopore sequencing generated SARS-CoV-2 whole genomes. Phylogenetic inference with maximum likelihood and Bayesian methods were used to determine lineages that seeded the local epidemic. Three patients were known to have travelled internationally and an outbreak was detected in a nearby supermarket. Sequencing of 50 samples produced 46 high-quality genomes. The sequences were classified as lineages: B, B.1, B.1.1.1, B.1.1.161, B.1.1.29, B.1.8, B.39, and B.40. All the sequences from persons under investigation (PUIs) in the supermarket outbreak (lineage B.1.8) fall within a clade from the Netherlands with good support (*p* > 0.9). In addition, a new mutation, 5209A>G, emerged within the Cape Town cluster. The molecular clock analysis suggests that this occurred around 13 March 2020 (95% confidence interval: 9–17 March). The phylogenetic reconstruction suggests at least nine early introductions of SARS-CoV-2 into Cape Town and an early localized transmission in a shopping environment. Genomic surveillance was successfully used to investigate and track the spread of early introductions of SARS-CoV-2 in Cape Town.

## 1. Introduction

Emerging infectious diseases have accompanied mankind for millennia. The first recorded pandemic, the plague of Athens, caused the death of ~100,000 people between 430–425 BCE [1]. Several more pandemics or large-scale epidemics have occurred throughout history. Infectious diseases of viral origin have increased dramatically since the turn of the 21st century, with the emergence of severe acute respiratory syndrome (SARS) in 2002 [2], influenza H1N1 in 2009 [3,4], chikungunya in 2014 [5], Zika in 2015 [6], and the ongoing coronavirus disease 2019 (COVID-19).

Since its origins in Wuhan, China, COVID-19 has spread rapidly across the globe, which is attributable to a globalized economy characterized by extensive international travel and commerce [1]. COVID-19 was declared a Public Health Emergency of International Concern (PHEIC) by the World Health Organization (WHO) on 31 January 2020, [7] and subsequently a pandemic on 11 March 2020 [8]. Intensified by high population density, SARS-CoV-2 spreads readily through human-to-human transmission as a respiratory pathogen. Outbreaks may initially go unnoticed because of infected individuals being infectious before symptom onset, as well as a substantial proportion developing no or only mild flu-like symptoms [9]. To date, SARS-CoV-2 has infected 117,660,021 people worldwide with 2,612,176 deaths (https://coronavirus.jhu.edu/map.html, accessed on 10 March 2021) [10].

South Africa recorded its first confirmed case on 5 March 2020 in KwaZulu-Natal (KZN) [11]. Subsequently, cases were reported in Gauteng (GP) and around the country, which prompted the government to announce a National State of Disaster on 15 March 2020. Shortly thereafter, the Western Cape (WC) experienced a rapid growth in the number of confirmed COVID-19 cases, with Cape Town as the epicenter. Cape Town is not only the oldest and second largest city in South Africa, with a population of 4,617,884 in the urban area (https://populationstat.com/south-africa/cape-town accessed 21 March 2021), but it is also the metropolitan municipality and economic center of the WC. By 26 October 2020, the WC had recorded a total of 115,002 confirmed COVID-19 cases, including 4325 deaths, with most cases reported in the City of Cape Town (*n* = 79,792 or 69.4%). These figures are a substantial undercount, as suspected cases in those younger than 55 years of age and without conditions predisposing to severe disease were not eligible for public sector testing for most of the pandemic. Of the seven health sub-districts within the Cape Town Metropole, the COVID-19 epicenter is in Tygerberg (*n* = 13,908) https://coronavirus.westerncape.gov.za/covid-19-dashboard, accessed 21 March 2021. Using a phylogenetic and epidemiological approach with nanopore sequencing technology, we investigated the introduction and timing of SARS-CoV-2 in the Cape Town Metropole.

## 2. Materials and Methods

### 2.1. Patient Sample Selection

Samples are routinely sent to the National Health Laboratory Service (NHLS) Virology Division at Tygerberg Academic Hospital for COVID-19 diagnostic testing. To identify the route of introduction of SARS-CoV-2 in Cape Town, we assessed 50 samples from the first confirmed cases in our laboratory between 9 March and 10 April 2020. These samples were selected based on their viral load (Ct value) and the availability of residual sample stored at −80 °C.

The project was approved by Stellenbosch University Human Research Ethics Committee (HREC) with reference number N20/04/008_COVID-19 and Project ID 14994.

### 2.2. Diagnostic Reverse Transcription Polymerase Chain Reaction (RT-qPCR)

Nucleic acid extraction was carried out using the NUCLISENS^®^ EASYMAG^®^ instrument (bioMerieux, Geneva, Switzerland) according to the manufacturer’s instructions. Isolated nucleic acid was stored at −80 °C. For diagnostic real-time PCR analyses, the Allplex™ 2019-nCoV Assay (Seegene Inc., Seoul, Korea) was used according to the manufacturer’s instructions. This assay targets a highly conserved region within the envelope (E) gene of viruses within the subgenus Sarbecovirus, as well as the SARS-CoV-2 nucleocapsid (N) and RNA-dependent RNA polymerase (RdRP) genes. For a subset of samples, an in-house PCR assay for the detection of the N/E-gene was used as described [12]. Ct values obtained were used as rough indicators of the viral loads of the samples. A positive control and non-template control were included in each PCR run.

### 2.3. Complementary DNA (cDNA) Synthesis and Tiling Polymerase Chain Reaction (PCR)

Residual RNA samples were retrieved from the NHLS, Tygerberg Virology Division. Superscript IV (Invitrogen, Marseille, France), with random hexamer primers, was used to produce complementary DNA (cDNA) according to the nCoV-2019 sequencing protocol made available by the ARTIC network (https://artic.network/ncov-2019, last accessed 21 March 2021)). The PCR tiling of COVID-19 virus protocol (PTC_9096_v109_revE_06Feb2020) and the Native Barcoding Kit (EXP-NBD104 and EXP-NBD11), provided by Oxford Nanopore Technologies (ONT), were used in conjunction with the ARTIC nCoV-2019 sequencing protocol [13]. Oligonucleotides (https://github.com/artic-network/artic-ncov2019/tree/master/primer_schemes/nCoV-2019/V3, last accessed 21 March 2021) were manufactured by Inqaba Biotech (Pretoria, South Africa). The recommended Q5^®^ Hot Start enzyme (NEB, Ipswich, MA, USA) was replaced with RANGER Mix (Bioline, Memphis, TN, USA) for the tiling PCR reactions due to availability. DNA quantity was assessed with the Qubit 2·0 fluorometer (Invitrogen, Carlsbad, CA, USA) using the Qubit™ dsDNA HS Assay Kit (Invitrogen, Carlsbad, CA, USA). To validate results, a non-template control was included from the cDNA step through to sequencing to ensure no cross-contamination occurred between steps.

### 2.4. Nanopore Sequencing and Data Analysis

The GridION electronic device (Oxford Nanopore Technologies, Oxford, UK) was used for sequencing, and the MinKNOW Release 19·12·6 software was set to either fast base calling or high-accuracy mode and run for up to twenty-four hours. FastQ files were exported from the GridION. Initially, sequences were assembled to NC_045512_3·1 in Geneious Prime 2021.0.3 (www.geneious.com, last accesses 21 March 2021) using Minimap2 version 2·17 [14]. To automate this process, assembly was carried out in Genome Detective 1·126 (https://www.genomedetective.com, last accessed 21 March 2021) [15] and the Coronavirus Typing Tool [16]. Readings mapped to the reference NC_045512_3·1 were polished, and low-quality mutations were filtered out using the bcftools 1·7-2 mpileup tool after genotype likelihood calculations. All mutations were validated by visualization of Binary Alignment Map (BAM) files using Geneious Prime 2021.0.3. This protocol follows the SARS-CoV-2 Genome Assembly Pipeline with Genome Detective [17].

Nextclade v0.14.0 (https://clades.nextstrain.org, last accessed 21 March 2021) [18] was used as quality assurance to report potential sequence quality issues, to identify differences between the Tygerberg sequences and the Wuhan-Hu-1 reference sequence, and to identify clades. Clades were assigned as defined by specific signature mutations [19]. To assign lineages, we used Phylogenetic Assignment of Named Global Outbreak Lineages or PANGO Lineages (Pangolin version v2.3.2, lineages version 21 February 2021) https://pangolin.cog-uk.io, last accesses 21 February 2021 [20]. A lineage is a geographically distinct cluster of sequences with evidence of ongoing transmission in that region. All the final edited consensus sequences were deposited in the Global initiative on sharing all influenza data, GISAID (https://www.gisaid.org, last accessed 21 March 2021) and the GISAID clade nomenclature was noted.

### 2.5. Phylogenetic Analysis

Tygerberg SARS-CoV-2 genotypes were analyzed against a backdrop of sequences from around the world. All the whole-genome sequences of SARS-CoV-2 were retrieved from the GISAID database as of 6 June 2020. Due to the size of this dataset, we randomly down sampled the dataset ten times to a total size of 3620 genotypes (acknowledged in Table S5). This subsample, along with 46 of the 47 genotypes from Cape Town, was analyzed on the NextStrain platform (https://nextstrain.org/ncov/global, last accessed 21 March 2021) [18] using the standard COVID-19 build, with slight modifications. Briefly, this build allows for the alignment of samples against one another using MAFFT [21] and Maximum Likelihood ML phylogenetic tree inference in IQ-TREE [22]. The build further uses TreeTime [23] to transfer the phylogeny into a time scaled tree topology at a constant clock rate of 8 × 10^−4^ mutations/site/year. TreeTime also performs an ancestral state reconstruction on the time-scaled tree topology. Essentially, this allowed us to reconstruct the spread of the global pandemic through time and space and to identify the time and most likely source of viral introductions into the Cape Town Metropole.2.6. BEAST Analysis.

Bayesian coalescent analyses were performed on clades 19A, 20A, and 20B of the NextStrain build to confirm the estimated date of origin for SARS-CoV-2 as proposed in recent literature [24], to infer the estimated date to the most recent common ancestor (MRCA) for major lineages, and to infer the estimated dates of viral introductions into Cape Town. Due to the large size of the five major lineages and clades, we randomly down sampled each dataset to ~200 taxa, while retaining all South African sequences. Down sampling was carried out to reduce the computational burden. Briefly, for each clade, sequences were aligned in MAFFT v 7 [21] and manually edited in Geneious Prime 2021.0.3 software (Biomatters Ltd., New Zealand). For each dataset, an ML-tree topology was inferred in IQ-Tree v 1·6·9 (GTR + G + I, with transfer support values). The resulting tree topologies were analyzed in TempEst [25] to ensure that the datasets contained enough diversity to fit a molecular clock.

Bayesian coalescent analyses were performed in BEAST v1·10 [26], under a strict molecular clock assumption and an exponential growth tree prior [27]. Runs were performed under both a fixed (at 8 × 10^−4^ substitutions per site per year) and relaxed clock rate. Markov chains were run in duplicate for a total of 100 million steps, with sampling every 10,000 steps in the chain. Runs were assessed in Tracer for sufficient convergence (Effective sample size (ESS) > 200) and maximum clade credibility trees were generated in TreeAnnotator after discarding 10% of runs as burn-in.

## 3. Results

### 3.1. Epidemiological and Demographic Information

**Figure 1:**
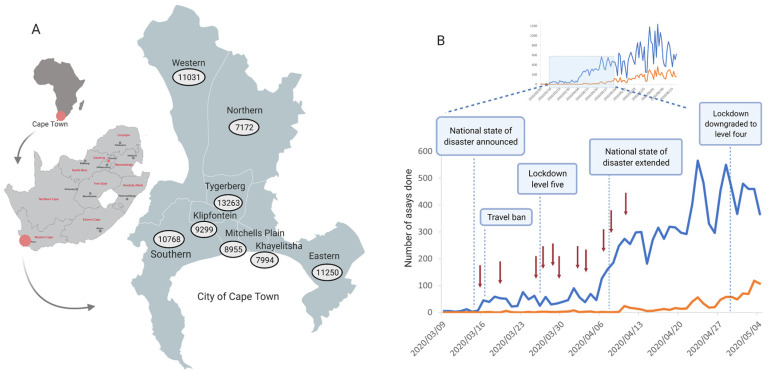
Epidemiology of SARS-CoV-2 in the Western Cape and the City of Cape Town. (**A**) Map of South Africa indicating the different provinces with a map of cases in the Cape Town Metropole on 31 July 2020 in the eight different health subdistricts. Data were obtained from the Western Cape Provincial Government, https://coronavirus.westerncape.gov.za, last accessed 21 March 2021. (**B**) Graphical presentation of the number of SARS-CoV-2 assays carried out and the number of positive cases at the Virology Division, NHLS, Tygerberg. The detailed view from 9 March 2020 indicates the timeline of the government response to the epidemic, with red arrows indicating early sample collection for sequencing at Tygerberg Hospital. Figure created using BioRender (https://biorender.com, last accessed 21 March 2021).

**Figure 2:**
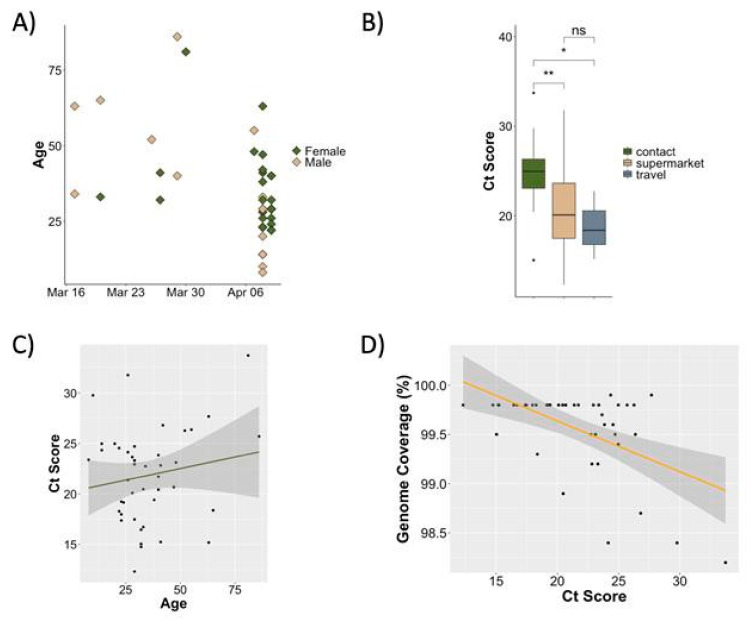
SARS-CoV-2 samples. (**A**) Scatter plot of samples obtained for this study showing sampling dates, age, and gender of patients. (**B**) Patient exposure status denoting whether patients had any travel history (grey), were part of a supermarket outbreak (brown) or were contacts of known cases (green). The Ct values may correlate with timing of sample collection. (**C**) The relationship between age of patients and Ct score. A *p*-value less than 0.05, is flagged with one star (*) and a *p*-value less than 0.01, is flagged with two stars (**) (**D**) The relationship between Ct score and resulting genome coverage after sequencing, showing higher overall genome coverage (hence sequence quality) from samples with lower Ct scores (higher viral loads).

The Tygerberg Virology Division started testing for SARS-CoV-2 on 9 March 2020. The first positive cases in Cape Town and Tygerberg were confirmed on 11 and 13 March 2020, respectively. The number of SARS-CoV-2 assays carried out and the number of positive cases at the Virology Division, NHLS, Tygerberg, are indicated in Figure 1. We received 545 samples up to 10 April 2020, of which 77 samples (14.1%) were positive and 50 (9.1%) were selected for sequencing. The age of the 50 patients whose samples were sequenced ranged from 8 to 86 years and included 34 women and 16 men (Figure 2). All the participants were South African citizens and three indicated recent international travel. All other participants were contacts of known positive cases, including 25 cases clustered in a supermarket. Demographic information is detailed in Supplementary Table S1.

### 3.2. Genome Sequencing and Phylogenetic Analysis

Of the 50 samples sequenced, 46 near-whole-genome sequences (>90% coverage, Supplementary Table S2) and one partial genome Tygerberg_23 were obtained with long stretches of NNNs (~15%), where the software is unable to define the bases. Three samples, including two samples that had high Ct values on diagnostic PCR (Tygerberg_39, Ct = 38; Tygerberg_40, Ct~37) failed to produce sequence data. Sequences are available from the GISAID database.

**Figure 3:**
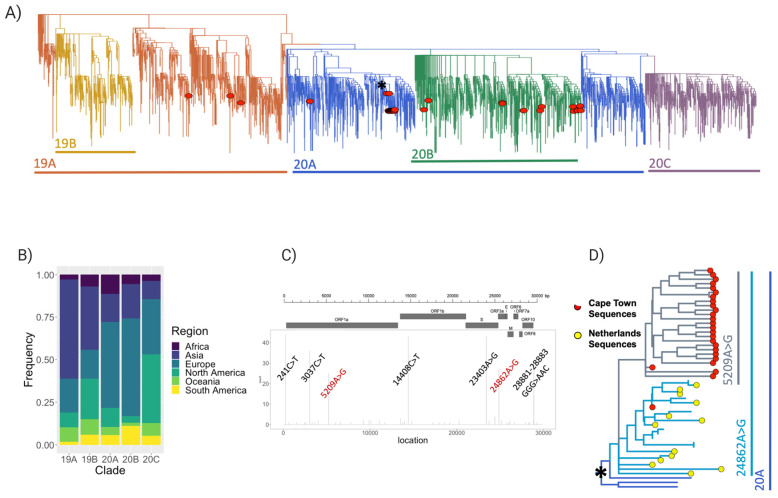
Phylogenetic and lineage analysis. (**A**) A time-scaled maximum-likelihood tree of 3620 sequences and 47 genotypes from Cape Town, Western Cape, South Africa. Major lineages of SARS-CoV-2 are labelled. (**B**) Stacked bar plot showing the lineage breakdown of the dataset by region, indicating the over-representation of lineage 20A (where most of the Cape Town sequences lie) with origins from Europe. (**C**) Genomic location and frequency of variants among the 47 genomes generated in this study mapped onto the genomic structure of SARS-CoV2, with common variants labelled. Variants labelled in red are observed either uniquely or at higher frequencies in our sequences compared to their global distribution. (**D**) Monophyletic cluster within the Cape Town sequences, showing closest divergence from isolates originating in the Netherlands, and the emergence of a Cape Town specific mutation within that cluster. This is a zoom-in of the tree in (A) at the position indicated by the asterisk (*).

To address our research questions, we performed a phylogenetic reconstruction of the Cape Town sequences (Figure 3A). The phylogenetic reconstruction containing 46 near-full-length genomes points toward at least nine introductions of SARS-CoV-2 into Cape Town. The observed genetic variants of SARS-CoV-2 in Cape Town can be divided into three main clades of the novel coronavirus: 19A (*n* = 3), 20A (*n* = 28), and 20B (*n* = 15) (Supplementary Table S3). These sequences were also classified in pangolin as lineages B (*n* = 1), B.1 (*n* = 2), B.1.1.1 (*n* = 2), B.1.1.161 (*n* = 1), B.1.1.29 (*n* = 12), B.1.8 (*n* = 27), B.39 (*n* = 1), and B.40 (*n* = 1). The classification of most of our sequences in the 20A and 20B clades or B.1.1.29 and B.1.8 lineages is an indication of their origins in Europe, where these clades are most represented (Figure 3B).

Our sequences averaged between 0 and 12 mutations (Supplementary Table S4), with seven mutation sites occurring at a high frequency, including two mutations (5209A>G and 24862A>G) occurring at a higher frequency in the Cape Town sequences than globally (Figure 3C). Twenty-five sequences sampled from a supermarket outbreak clustered together. A closer look at this cluster suggests an introduction from the Netherlands, with the 24862A>G mutation being inherited from the Netherlands lineage and 5209A>G emerging within the Cape Town cluster (Figure 3D).

### 3.3. Timing and Possible Source of Infection

Bayesian analyses were performed to investigate the timing and possible source of the introductory events observed. Based on the full phylogenetic tree, we estimated a mean mutation rate of ~25 substitutions per genome, per year. For a virus with a genome of ~30 kbp, this roughly translates to a mutation rate of 0.0008 substitutions/site/year. Due to the overall low genetic diversity of SARS-CoV-2, the posterior support for splits in Bayesian trees were not well-supported. Furthermore, because of the downsampling of the datasets, the ancestral state reconstruction will be different to that observed in the Nextstrain build.

**Figure 4:**
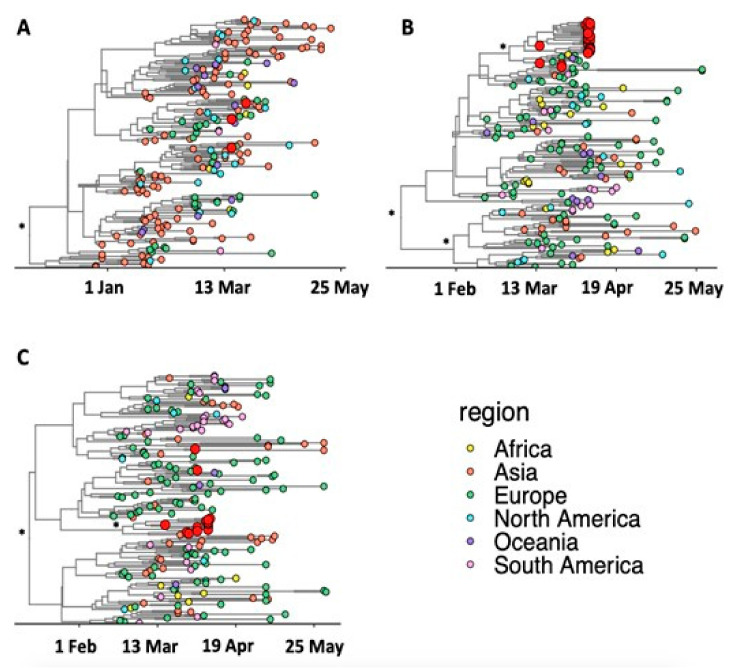
Bayesian Maximum Clade Credibility trees of major SARS-CoV-2 clades. (**A**) Clade 19A, (**B**) Clade 20 A and (**C**) Clade 20B. The sequences are colored according to their region of sampling, while Cape Town sequences are labelled in red. The branches of the tree topology are in calendar time while well-supported splits in the tree topology (posterior support > 0.9) are marked with an asterisk (*).

All the introductions appear to have occurred between 17 February and 25 March, with the bulk of introductions during the first two weeks of March. Of the three 19A isolates (Figure 4A), two samples, Tygerberg_06 (lineage B.40) and Tygerberg_31 (lineage B.39), clustered in a large European clade with strong posterior support (*p* > 0.9). The clustering for Tygerberg_04 (lineage B) is less certain in the tree. This could possibly be due to the lack of genetic diversity from the Wuhan reference strain. Tygerberg_04 had no mutations, which increases the uncertainty of the placement of this isolate in the Bayesian phylogenetic tree. The molecular clock analyses suggest an introduction of these three isolates at sometime between the last week of February and the end of the first week of March 2020. Of the isolates that were classified as belonging to clade 20A or pangolin lineage B.1.1 (Figure 4B), samples Tygerberg_02 and Tygerberg_03 clustered together with a sample from the United Kingdom (U.K.), with good posterior support (*p* > 0.9).

The supermarket outbreak formed a large monophyletic cluster (lineage B.1.8) rooted in a clade from the Netherlands with strong support (*p* > 0.9). The molecular clock analysis in BEAST suggests that these two introductions occurred within a small timeframe of one another (~13 March; 95% confidence interval: 9–17 March). Based on the molecular clock analyses, we estimate that the clade 20B (lineages B.1.1.1, B.1.1.29 and B.1.1.161) (Figure 4C) cluster must have been introduced sometime between 17 February and 24 March. The large range in the inferred dates is principally a result of the low diversity in these early sequences, which increases the uncertainty. Due to the low diversity of the sequences in this clade and the slow mutation rate of SARS-CoV-2, the eleven sequences that we believe to be associated with one another are all dispersed throughout the sub-tree.

## 4. Discussion

SARS-CoV-2 has been detected world-wide, with 720,780 virus sequences available in GISAID (https://www.gisaid.org, accessed on 9 March 2021). The unprecedented number of sequences can be used to investigate SARS-CoV-2 genetic diversity and mutations [28]. A dynamic nomenclature system for SARS-CoV-2 can be used to track the lineages as they emerge and move in local and global patterns [19]. Phylogenetic and phylogeographic methods were used to track the early emergence of the virus in Italy [29,30]; New York [31]; and Pernambuco, Brazil [32]. In addition to tree inference, epidemic and sequence simulation methods established the earliest sustained transmission networks in Europe and the USA [33].

Our phylogenetic reconstruction contains 46 near-complete genomes sampled during the first month of the epidemic in Cape Town, South Africa. This correlates to 59.7% of the cases diagnosed during this time-period in Tygerberg Health District, 9.1% in Cape Town, and 7.5% in the Western Cape Province. The phylogenetic reconstruction suggests at least nine early introductions of SARS-CoV-2 into Cape Town and an early localized transmission in a working environment. 

Three isolates (Tygerberg_04, Tygerberg_06, and Tygerberg_31) contain mutations closer to the Asian variants of SARS-CoV-2, clustering in the 19A clade or PANGO lineages B, B.39, and B.40. The genetic sequence of Tygerberg_04 (lineage B) does not differ from the Wuhan-Hu-1 reference strain, the first genome sequence of SARS-CoV-2 published in early January 2020 [34]. Contact tracing indicated recent travel history to the U.K. for the individual from whom this virus genotype was obtained. This suggests transmission from China to the U.K. and onwards to Cape Town. The base of lineage B lies in China, with extensive global spread [19]. Two mutations, 8782T>C and 28144C>T, define this lineage (https://cov-lineages.org/lineages/lineage_B.html, accessed 21 March 2021).

The Tygerberg_06 (lineage B.40) and Tygerberg_31 (lineage B.39) genotypes share two common mutations (26144G>T and 14805C>T) in relation to the Wuhan-Hu-1 reference stain. For Tygerberg_06, the molecular clock and ancestral state reconstruction suggest spread from China to the UK and onward to Cape Town. Along the chain of transmission, additional mutation events occurred leading to the genetic profile observed in Tygerberg_06. For Tygerberg_31, the most likely route of transmission was from China to Australia, then from Australia to the United States (defined by the 17247T>C mutation), and then onward to Cape Town.

The remainder of the Cape Town sequences were classified into Nextstrain clade 20A or pangolin lineages B.1 (*n* = 1) and B.1.8 (*n* = 27); and clade 20B; or lineages B.1.1.1, B.1.1.161, (*n* = 1 each), and B.1.1.29 (*n* = 12). The 20A–20C clades of SARS-CoV-2 are mainly associated with infections outside of Europe. The 20A clade contains 28 genotypes from Cape Town that are the result of two separate introductions. The reconstruction suggests transmission of a Spanish variant (20268A>G) to the U.K. and onward to Cape Town, giving rise to the genetic profile of Tygerberg_02 and Tygerberg_03. Contact tracing confirms these two individuals were contacts of a South African traveler who had recently returned from the U.K.

The second 20A introduction was Tygerberg_05. Contact tracing suggest this was a Dutch variant of SARS-CoV-2 acquired while travelling in the Netherlands. The Dutch variant is defined by the common mutation 24862A>G relative to the Wuhan reference strain. From the Netherlands, this variant spread to several countries (New Zealand, Colombia, and Austria). This cluster also contained another sequence from South Africa (R07601) sampled in the northern province of Limpopo (LP) (GISAID EPI_ISL_450300). This suggests multiple introductions of the Dutch variant into the country or local spread from the introduction to Cape Town. South Africa and the Cape share deep cultural and historic connections with the Netherlands, with frequent travel between the two countries. Following the introduction of the Dutch variant into Cape Town, the virus further diversified with the acquisition of an additional mutation 5209A>G before causing a large outbreak in a supermarket (supermarkets were among the few essential services allowed to continue operating during lockdown level 5 in South Africa). This variant, with a rare mutation 5209A>G, has been observed only in the supermarket outbreak in Cape Town. Keeping track of unique mutations like this will allow us to understand how the virus spreads between different locations in the city, the surrounding countryside and throughout the country. This is the primary objective of Network for Genomic Surveillance in South Africa (NGS-SA) [35].

Fourteen of the Cape Town sequences clustered in clade 20B or pangolin lineages B.1.1.1, B.1.1.161, and B.1.1.29. We think these fourteen sequences are the result of at least four unique introductions. Tygerberg_29 (lineage B.1.1.161) and Tygerberg_43 (lineage B.1.1.29) appear to be unique introductions from the U.K. and the Netherlands, respectively. Tygerberg_30 and Tygerberg_32, both lineage B.1.1.1, appear to be due to a unique introduction of a U.K. variant (defined by the common mutation 10097G>A). The remaining 11 Tygerberg sequences cluster intermittently in a clade rooted in Italy. The mutation profiles of these 11 sequences are so close to one another that it is difficult to distinguish whether they are the result of a single introduction or due to multiple introductions into Cape Town. However, due to their close genetic similarity, we classified this as a single introduction. Low genetic diversity of SARS-CoV-2 in the dataset may influence the accuracy of phylogenetic inference and be a limitation in the study. However, genomic and epidemiological data provide unique insights into the spread and transmission of the early SARS-CoV-2 epidemic to Cape Town, and indicate the presence of most of the initial clades and lineages of SARS-CoV-2 as defined by specific signature mutations [19] (https://cov-lineages.org, accessed 21 March 2021).

## 5. Conclusions

In conclusion, South Africa was one of the first countries in Africa to set up genomics surveillance of SARS-CoV-2. Based on the clade and lineage data, we confirmed that SARS-CoV-2 was introduced into Cape Town by multiple introductory events. Molecular clock analyses showed that all the introductions occurred during the last week of February and the first two weeks of March 2020. Subsequent local transmission in a supermarket cluster showed that a new lineage with a specific mutation 5209A>G was first identified in Cape Town.

## Data Availability

Sequences are available via the GISAID database.

## Supplementary Materials

The following are available online at https://www.mdpi.com/1999-4915/13/3/526/s1, Table S1: Demographic information with Ct values, Table S2: Genome coverage and genome detective results for 47 sequences, Table S3: GISAID reference ID, clade, and lineage information, Table S4: Total number and list of sequence mutations, Table S5: GISAID acknowledgements.

## References

[1] Morens D.M., Daszak P., Markel H., Taubenberger J.K. (2020). Pandemic COVID-19 Joins History’s Pandemic Legion. mBio.

[2] Drosten C., Günther S., Preiser W., van der Werf S., Brodt H.R., Becker S., Rabenau H., Panning M., Kolesnikova L., Fouchier R.A. (2003). Identification of a novel coronavirus in patients with severe acute respiratory syndrome. N. Engl. J. Med..

[3] Dawood F.S., Jain S., Finelli L., Shaw M.W., Lindstrom S., Garten R.J., Gubareva L.V., Xu X., Bridges C.B., Novel Swine-Origin Influenza A (H1N1) Virus Investigation Team (2009). Emergence of a novel swine-origin influenza A (H1N1) virus in humans. N. Engl. J. Med..

[4] Smith G.J., Vijaykrishna D., Bahl J., Lycett S.J., Worobey M., Pybus O.G., Ma S.K., Cheung C.L., Raghwani J., Bhatt S. (2009). Origins and evolutionary genomics of the 2009 swine-origin H1N1 influenza A epidemic. Nature.

[5] Faria R.N., Lourenço J., Marques de Cerqueira E., Maia de Lima M., Pybus O., Carlos Junior Alcantara L. (2016). Epidemiology of Chikungunya Virus in Bahia, Brazil, 2014–2015. PLoS Curr..

[6] Faria N.R., Azevedo R.D.S.D.S., Kraemer M.U.G., Souza R., Cunha M.S., Hill S.C., Thézé J., Bonsall M.B., Bowden T.A., Rissanen I. (2016). Zika virus in the Americas: Early epidemiological and genetic findings. Science.

[7] World Health Organization (2020). WHO. Novel Coronavirus (2019-nCoV). Situation Report—1.

[8] World Health Organization (2020). WHO. Novel Coronavirus (2019-nCoV). Situation Report—51.

[9] Li R., Pei S., Chen B., Song Y., Zhang T., Yang W., Shaman J. (2020). Substantial undocumented infection facilitates the rapid dissemination of novel coronavirus (SARS-CoV-2). Science.

[10] Dong E., Du H., Gardner L. (2020). An interactive web-based dashboard to track COVID-19 in real time. Lancet Infect Dis..

[11] Giandhari J., Pillay S., Wilkinson E., Tegally H., Sinayskiy I., Schuld M., Lourenço J., Chimukangara B., Lessells R., Moosa Y. (2020). Early transmission of SARS-CoV-2 in South Africa: An epidemiological and phylogenetic report. Int. J. Infect. Dis..

[12] Corman V.M., Landt O., Kaiser M., Molenkamp R., Meijer A., Chu D.K., Bleicker T., Brünink S., Schneider J., Schmidt M.L. (2020). Detection of 2019 novel coronavirus (2019-nCoV) by real-time RT-PCR. Eurosurveillance.

[13] Quick J. (2020). nCoV-2019 Sequencing Protocol. protocols.io.

[14] Li H. (2018). Minimap2: Pairwise alignment for nucleotide sequences. Bioinformatics.

[15] Vilsker M., Moosa Y., Nooij S., Fonseca V., Ghysens Y., Dumon K., Pauwels R., Alcantara L.C., Vanden Eynden E., Vandamme A.M. (2019). Genome Detective: An automated system for virus identification from high-throughput sequencing data. Bioinformatics.

[16] Cleemput S., Dumon W., Fonseca V., Abdool Karim W., Giovanetti M., Alcantara L.C., Deforche K., de Oliveira T. (2020). Genome Detective Coronavirus Typing Tool for rapid identification and characterization of novel coronavirus genomes. Bioinformatics.

[17] Pillay S., Giandhari J., Tegally H., Wilkinson E., Chimukangara B., Lessells R., Moosa Y., Mattison S., Gazy I., Fish M. (2020). Whole Genome Sequencing of SARS-CoV-2: Adapting Illumina Protocols for Quick and Accurate Outbreak Investigation during a Pandemic. Genes.

[18] Hadfield J., Megill C., Bell S.M., Huddleston J., Potter B., Callender C., Sagulenko P., Bedford T., Neher R.A. (2018). Nextstrain: Real-time tracking of pathogen evolution. Bioinformatics.

[19] Rambaut A., Holmes E.C., O’Toole Á., Hill V., McCrone J.T., Ruis C., du Plessis L., Pybus O.G. (2020). A dynamic nomenclature proposal for SARS-CoV-2 lineages to assist genomic epidemiology. Nat. Microbiol..

[20] (2020). Pangolin was Created by Áine O’Toole, JT McCrone and Emily Scher. It Uses Lineages from Rambaut et al. https://github.com/cov-lineages/pangolin.

[21] Katoh K., Standley D.M. (2013). MAFFT multiple sequence alignment software version 7: Improvements in performance and usability. Mol. Biol. Evol..

[22] Nguyen L.T., Schmidt H.A., von Haeseler A., Minh B.Q. (2015). IQ-TREE: A fast and effective stochastic algorithm for estimating maximum-likelihood phylogenies. Mol. Biol. Evol..

[23] Sagulenko P., Puller V., Neher R.A. (2018). TreeTime: Maximum likelihood phylodynamic analysis. Virus Evol..

[24] Duchene S., Featherstone L., Haritopoulou-Sinanidou M., Rambaut A., Lemey P., Baele G. (2020). Temporal signal and the phylodynamic threshold of SARS-CoV-2. Virus Evol..

[25] Rambaut A., Lam T.T., Max Carvalho L., Pybus O.G. (2016). Exploring the temporal structure of heterochronous sequences using TempEst (formerly Path-O-Gen). Virus Evol..

[26] Suchard M.A., Lemey P., Baele G., Ayres D.L., Drummond A.J., Rambaut A. (2018). Bayesian phylogenetic and phylodynamic data integration using BEAST 1.10. Virus Evol..

[27] Drummond A.J., Nicholls G.K., Rodrigo A.G., Solomon W. (2002). Estimating mutation parameters, population history and genealogy simultaneously from temporally spaced sequence data. Genetics.

[28] Van Dorp L., Acman M., Richard D., Shaw L.P., Ford C.E., Ormond L., Owen C.J., Pang J., Tan C.C.S., Boshier F.A.T. (2020). Emergence of genomic diversity and recurrent mutations in SARS-CoV-2. Infect. Genet. Evol..

[29] Stefanelli P., Faggioni G., Lo Presti A., Fiore S., Marchi A., Benedetti E., Fabiani C., Anselmo A., Ciammaruconi A., Fortunato A. (2020). Whole genome and phylogenetic analysis of two SARS-CoV-2 strains isolated in Italy in January and February 2020: Additional clues on multiple introductions and further circulation in Europe. Eurosurveillance.

[30] Lai A., Bergna A., Caucci S., Clementi N., Vicenti I., Dragoni F., Cattelan A.M., Menzo S., Pan A., Callegaro A. (2020). Molecular Tracing of SARS-CoV-2 in Italy in the First Three Months of the Epidemic. Viruses.

[31] Maurano M.T., Ramaswami S., Zappile P., Dimartino D., Boytard L., Ribeiro-Dos-Santos A.M., Vulpescu N.A., Westby G., Shen G., Feng X. (2020). Sequencing identifies multiple early introductions of SARS-CoV-2 to the New York City region. Genome Res..

[32] Paiva M.H.S., Guedes D.R.D., Docena C., Bezerra M.F., Dezordi F.Z., Machado L.C., Krokovsky L., Helvecio E., da Silva A.F., Vasconcelos L.R.S. (2020). Multiple Introductions Followed by Ongoing Community Spread of SARS-CoV-2 at One of the Largest Metropolitan Areas of Northeast Brazil. Viruses.

[33] Worobey M., Pekar J., Larsen B.B., Nelson M.I., Hill V., Joy J.B., Rambaut A., Suchard M.A., Wertheim J.O., Lemey P. (2020). The emergence of SARS-CoV-2 in Europe and North America. Science.

[34] Zhou P., Yang X.L., Wang X.G., Hu B., Zhang L., Zhang W., Si H.R., Zhu Y., Li B., Huang C.L. (2020). A pneumonia outbreak associated with a new coronavirus of probable bat origin. Nature.

[35] Msomi N., Mlisana K., de Oliveira T., Network for Genomic Surveillance in South Africa Writing Group (2020). A genomics network established to respond rapidly to public health threats in South Africa. Lancet Microbe.

