# Supplementary material for: Multiple Early Introductions of SARS-CoV-2 to Cape Town, South Africa
Source: Viruses. 2021 Mar 22;13(3):526. doi: 10.3390/v13030526 (PMC8005015; doi:10.3390/v13030526)
Supplement: Supplementary file 1 [file viruses-13-00526-s001.zip › Engelbrecht_supplementary_Tables.pdf]

Supplementary Table 1. Demographic information and Ct value

Sequence Name	Collection Date	Gender	Age	Health Sub-district	Ct E gene	Ct RdRp	Ct N gene
Tygerberg_01	2020-03-17	Male*	63	Southern	12·40	16·11	17·01
Tygerberg_02	2020-03-27	Female	41	Tygerberg	21·19	22·48	24·78
Tygerberg_03	2020-03-27	Female	32	Tygerberg	12·66	15·15	17·30
Tygerberg_04	2020-03-20	Male**	65	Southern	16·89	18·33	19·88
Tygerberg_05	2020-03-17	Male***	34	Tygerberg	18·73	25·46	24·01
Tygerberg_06	2020-03-20	Female	33	Tygerberg	18·67	20·59	22·12
Tygerberg_07	2020-04-08	Female	23	Tygerberg	15·00	17·90	19·20
Tygerberg_08	2020-04-08	Female	28	Tygerberg	20·09[#]	None	None
Tygerberg_09	2020-04-08	Female	29	Tygerberg	20·90	22·50	25·40
Tygerberg_10	2020-04-08	Female	41	Tygerberg	13·10	14·80	17·80
Tygerberg_11	2020-04-07	Female	50	Northern	21·30	22·70	25·30
Tygerberg_12	2020-04-08	Female	23	Tygerberg	17·20	19·30	21·20
Tygerberg_13	2020-04-08	Female	42	Tygerberg	24·60	26·20	29·60
Tygerberg_14	2020-04-08	Female	28	Tygerberg	21·80	23·10	26·00
Tygerberg_15	2020-04-08	Male	33	Tygerberg	14·50	16·60	19·00
Tygerberg_16	2020-04-08	Female	38	Tygerberg	17·10	19·00	22·10
Tygerberg_17	2020-04-08	Female	32	Tygerberg	13·90	16·40	19·00
Tygerberg_18	2020-04-08	Female	23	Tygerberg	15·60	17·70	20·60
Tygerberg_19	2020-04-08	Male	29	Tygerberg	9·80	12·80	14·30
Tygerberg_20	2020-04-09	Male	29	Northern	14·90	17·50	20·00
Tygerberg_21	2020-04-09	Female	26	Northern	19·50	20·90	23·70
Tygerberg_22	2020-04-09	Female	22	Northern	16·70	18·50	19·60
Tygerberg_23	2020-04-09	Female	40	Northern	20·00	21·70	23·40
Tygerberg_24	2020-04-09	Female	40	Northern	21·90	24·10	25·50
Tygerberg_25	2020-04-09	Female	29	Northern	22·90	24·70	26·50
Tygerberg_26	2020-04-09	Female	24	Northern	17·60	19·20	20·60
Tygerberg_27	2020-04-09	Female	32	Northern	12·10	15·20	16·90
Tygerberg_28	2020-04-09	Female	22	Northern	22·70	24·50	26·40
Tygerberg_29	2020-03-26	Male	52	Tygerberg	26·26[#]	None	None
Tygerberg_30	2020-03-29	Male	86	Tygerberg	23·93	25·17	27·95
Tygerberg_31	2020-03-29	Male	40	Tygerberg	18·89	19·72	22·64
Tygerberg_32	2020-03-30	Female	81	Tygerberg	30·63	33·41	37·12
Tygerberg_33	2020-04-02	Male	30	Tygerberg	Unknown	Unknown	Unknown
Tygerberg_34	2020-04-03	Male	59	Tygerberg	Unknown	Unknown	Unknown
Tygerberg_35	2020-04-03	Female	58	Tygerberg	Unknown	Unknown	Unknown
Tygerberg_36	2020-04-03	Female	25	Tygerberg	Unknown	Unknown	Unknown
Tygerberg_37	2020-04-09	Female	26	Northern	22·20	24·10	26·10

Tygerberg_38	2020-04-09	Female	47	Northern	19·10	20·50	22·40
Tygerberg_39	2020-04-09	Female	41	Northern	38·18[#]	None	None
Tygerberg_41	2020-04-08	Male	10	Eastern	28·40	29·20	31·70
Tygerberg_42	2020-04-08	Male	8	Eastern	21·80	23·50	24·80
Tygerberg_43	2020-04-07	Male	55	Eastern	24·70	26·50	27·90
Tygerberg_44	2020-04-09	Female	29	Eastern	21·60	23·50	24·80
Tygerberg_45	2020-04-08	Female	14	Eastern	22·90	24·60	27·40
Tygerberg_46	2020-04-08	Female	63	Eastern	25·90	28·10	29·00
Tygerberg_47	2020-04-08	Male	14	Eastern	22·40	24·40	26·20
Tygerberg_48	2020-04-08	Male	20	Eastern	23·20	25·00	26·70
Tygerberg_49	2020-04-08	Female	44	Eastern	33·80	37·00	37·30
Tygerberg_50	2020-04-08	Female	26	Tygerberg	29·50	31·10	34·70

*	SA: travel to UK
**	SA: travel to UK
***	SA: travel to Netherlands
#	in house PCR

Supplementary Table 2. Genome coverage and Genome Detective results for 47 sequences

Sequence Name	Begin	End	Coverage	Score	Concordance	Matches	Identities	I/D/M/F
Tygerberg_01	31	29866	99·80%	59648	99·90%	29836 (100%)	29830 (99·9%)	0/0
Tygerberg_02	31	29686	99·20%	59288	99·90%	29656 (100%)	29650 (99·9%)	0/0
Tygerberg_03	31	29776	99·50%	59468	99·90%	29746 (100%)	29740 (99·9%)	0/0
Tygerberg_04	31	29379	96·50%	57688	99·90%	28847 (100%)	28844 (99·9%)	0/0
Tygerberg_05	31	29380	97·30%	58180	99·90%	29100 (100%)	29095 (99·9%)	0/0
Tygerberg_06	31	29380	94·60%	56570	99·90%	28297 (100%)	28291 (99·9%)	0/0
Tygerberg_07	31	29869	99·80%	59650	99·90%	29839 (100%)	29832 (99·9%)	0/0
Tygerberg_08	31	29866	99·80%	59648	99·90%	29836 (100%)	29830 (99·9%)	0/0
Tygerberg_09	31	29662	98·20%	58714	99·90%	29369 (100%)	29363 (99·9%)	0/0
Tygerberg_10	31	29874	99·80%	59656	99·90%	29844 (100%)	29836 (99·9%)	0/0
Tygerberg_11	31	29394	97·40%	58206	99·90%	29115 (100%)	29109 (99·9%)	0/0
Tygerberg_12	31	29867	99·80%	59646	99·90%	29837 (100%)	29830 (99·9%)	0/0
Tygerberg_13	31	29380	92·70%	55428	99·90%	27724 (100%)	27719 (99·9%)	0/0
Tygerberg_14	31	29471	97·30%	58146	99·90%	29087 (100%)	29080 (99·9%)	0/0
Tygerberg_15	31	29873	99·80%	59662	99·90%	29843 (100%)	29837 (99·9%)	0/0
Tygerberg_16	31	29849	99·70%	59614	99·90%	29819 (100%)	29813 (99·9%)	0/0
Tygerberg_17	31	29856	99·70%	59628	99·90%	29826 (100%)	29820 (99·9%)	0/0
Tygerberg_18	31	29866	99·80%	59644	99·90%	29836 (100%)	29829 (99·9%)	0/0
Tygerberg_19	31	29877	99·80%	59666	99·90%	29847 (100%)	29840 (99·9%)	0/0
Tygerberg_20	31	29874	99·80%	59656	99·90%	29844 (100%)	29836 (99·9%)	0/0
Tygerberg_21	31	29867	99·80%	59646	99·90%	29837 (100%)	29830 (99·9%)	0/0
Tygerberg_22	31	29867	99·80%	59646	99·90%	29837 (100%)	29830 (99·9%)	0/0
Tygerberg_23	19	29614	83·90%	50138	99·90%	25077 (100%)	25073 (99·9%)	0/0

Tygerberg_24	30	29667	98·20%	58730	99·90%	29377<br>(100%)	29371<br>(99·9%)	0/0
Tygerberg_25	31	29453	97·60%	58324	99·90%	29174<br>(100%)	29168<br>(99·9%)	0/0
Tygerberg_26	31	29866	99·80%	59648	99·90%	29836<br>(100%)	29830<br>(99·9%)	0/0
Tygerberg_27	31	29874	99·80%	59652	99·90%	29844<br>(100%)	29835<br>(99·9%)	0/0
Tygerberg_28	31	29676	98·20%	58670	99·90%	29351<br>(100%)	29341<br>(99·9%)	0/0
Tygerberg_29	31	29382	97·30%	58178	99·90%	29103<br>(100%)	29096<br>(99·9%)	0/0
Tygerberg_30	31	29381	96·30%	57562	99·90%	28805<br>(100%)	28793<br>(99·9%)	0/0
Tygerberg_31	31	29455	98·00%	58574	99·90%	29299<br>(100%)	29293<br>(99·9%)	0/0
Tygerberg_32	31	29381	90·40%	54016	99·90%	27030<br>(100%)	27019<br>(99·9%)	0/0
Tygerberg_33	31	29380	94·90%	56750	99·90%	28389<br>(100%)	28382<br>(99·9%)	0/0
Tygerberg_34	31	29783	99·50%	59470	99·90%	29753<br>(100%)	29744<br>(99·9%)	0/0
Tygerberg_35	31	29381	92·20%	55096	99·90%	27560<br>(100%)	27554<br>(99·9%)	0/0
Tygerberg_36	31	29690	98·00%	58594	99·90%	29311<br>(100%)	29304<br>(99·9%)	0/0
Tygerberg_37	31	29381	92·80%	55450	99·90%	27737<br>(100%)	27731<br>(99·9%)	0/0
Tygerberg_38	31	29395	96·90%	57938	99·90%	28981<br>(100%)	28975<br>(99·9%)	0/0
Tygerberg_41	31	29384	90·80%	54264	99·90%	27148<br>(100%)	27140<br>(99·9%)	0/0
Tygerberg_42	31	29386	96·50%	57682	99·90%	28855<br>(100%)	28848<br>(99·9%)	0/0
Tygerberg_43	12	29381	92·80%	55478	99·90%	27753<br>(100%)	27746<br>(99·9%)	0/0
Tygerberg_44	31	29705	99·20%	59310	99·90%	29675<br>(100%)	29665<br>(99·9%)	0/0
Tygerberg_45	31	29383	97·30%	58182	99·90%	29105<br>(100%)	29098<br>(99·9%)	0/0
Tygerberg_46	11	29877	99·90%	59686	99·90%	29867<br>(100%)	29855<br>(99·9%)	0/0
Tygerberg_47	31	29756	97·80%	58468	99·90%	29248<br>(100%)	29241<br>(99·9%)	0/0
Tygerberg_48	31	29866	99·80%	59620	99·90%	29836<br>(99.9%)	29826<br>(99·9%)	3/0
Tygerberg_50	31	29384	91·80%	54882	99·90%	27451<br>(100%)	27446<br>(99·9%)	0/0

Supplementary Table 3. GISAID Reference ID, Clade, and lineage information

Sample_ID	GISAID ID	Collection Date	GISAID Clade	Next clade	Pangolin Lineage
Tygerberg_01	EPI_ISL_464112	2020-03-17	G	20A	B·1·8
Tygerberg_02	EPI_ISL_464113	2020-03-27	G	20A	B·1·5
Tygerberg_03	EPI_ISL_464114	2020-03-27	G	20A	B·1·5
Tygerberg_04	EPI_ISL_464115	2020-03-20	L	19A	B
Tygerberg_05	EPI_ISL_464116	2020-03-17	G	20A	B·1·8
Tygerberg_06	EPI_ISL_464117	2020-03-20	V	19A	B·2·1
Tygerberg_07	EPI_ISL_464118	2020-04-08	G	20A	B·1·8
Tygerberg_08	EPI_ISL_464119	2020-04-08	G	20A	B·1·8
Tygerberg_09	EPI_ISL_464120	2020-04-08	G	20A	B·1·8
Tygerberg_10	EPI_ISL_464121	2020-04-08	G	20A	B·1·8
Tygerberg_11	EPI_ISL_464122	2020-04-07	G	20A	B·1·8
Tygerberg_12	EPI_ISL_464123	2020-04-08	G	20A	B·1·8
Tygerberg_13	EPI_ISL_464124	2020-04-08	G	20A	B·1·8
Tygerberg_14	EPI_ISL_464125	2020-04-08	G	20A	B·1·8
Tygerberg_15	EPI_ISL_464126	2020-04-08	G	20A	B·1·8
Tygerberg_16	EPI_ISL_464127	2020-04-08	G	20A	B·1·8
Tygerberg_17	EPI_ISL_464128	2020-04-08	G	20A	B·1·8
Tygerberg_18	EPI_ISL_464129	2020-04-08	G	20A	B·1·8
Tygerberg_19	EPI_ISL_464130	2020-04-08	G	20A	B·1·8
Tygerberg_20	EPI_ISL_464131	2020-04-09	G	20A	B·1·8
Tygerberg_21	EPI_ISL_464132	2020-04-09	G	20A	B·1·8
Tygerberg_22	EPI_ISL_464133	2020-04-09	G	20A	B·1·8
Tygerberg_23	EPI_ISL_464134	2020-04-09	G	20A	B·1·5
Tygerberg_24	EPI_ISL_464135	2020-04-09	G	20A	B·1·8
Tygerberg_25	EPI_ISL_464136	2020-04-09	G	20A	B·1·8
Tygerberg_26	EPI_ISL_464137	2020-04-09	G	20A	B·1·8
Tygerberg_27	EPI_ISL_464138	2020-04-09	G	20A	B·1·8
Tygerberg_28	EPI_ISL_464139	2020-04-09	G	20A	B·1·8
Tygerberg_29	EPI_ISL_464140	2020-03-26	Other	20B	B·1·1
Tygerberg_30	EPI_ISL_464141	2020-03-29	GR	20B	B·1·1·1
Tygerberg_31	EPI_ISL_464142	2020-03-29	Other	19A	B·2·2
Tygerberg_32	EPI_ISL_464143	2020-03-30	GR	20B	B·1·1·1
Tygerberg_33	EPI_ISL_464144	2020-04-02	GR	20B	B·1·1
Tygerberg_34	EPI_ISL_464145	2020-04-03	GR	20B	B·1·1
Tygerberg_35	EPI_ISL_464146	2020-04-03	GR	20B	B·1·1
Tygerberg_36	EPI_ISL_464147	2020-04-03	GR	20B	B·1·1
Tygerberg_37	EPI_ISL_464148	2020-04-09	G	20A	B·1·8
Tygerberg_38	EPI_ISL_464149	2020-04-09	G	20A	B·1·8
Tygerberg_41	EPI_ISL_464150	2020-04-08	GR	20B	B·1·1·26
Tygerberg_42	EPI_ISL_464151	2020-04-08	GR	20B	B·1·1

Tygerberg_43	EPI_ISL_464152	2020-04-07	GR	20B	B·1·1
Tygerberg_44	EPI_ISL_464153	2020-04-09	GR	20B	B·1·1
Tygerberg_45	EPI_ISL_464154	2020-04-08	GR	20B	B·1·1
Tygerberg_46	EPI_ISL_464155	2020-04-08	GR	20B	B·1·1
Tygerberg_47	EPI_ISL_464156	2020-04-08	GR	20B	B·1·1
Tygerberg_48	EPI_ISL_464157	2020-04-08	GR	20B	B·1·1
Tygerberg_50	EPI_ISL_464158	2020-04-08	G	20A	B·1·8

Supplementary Table 4. Total number and mutations of 47 sequences

Sample_ID	Total Mutations	Mutations
Tygerberg_01	6	241C>T, 3037C>T, 5209A>G, 14408C>T, 23403A>G, 24862A>G
Tygerberg_02	6	241C>T, 3037C>T, 14408C>T, 20268A>G, 23403A>G, 28854C>T
Tygerberg_03	6	241C>T, 3037C>T, 14408C>T, 20268A>G, 23403A>G, 28854C>T
Tygerberg_04	0	None
Tygerberg_05	5	241C>T, 3037C>T, 14408C>T, 23403A>G, 24862A>G
Tygerberg_06	6	2480A>G, 2558C>T, 11083G>T, 14805C>T, 17977C>T, 26144G>T
Tygerberg_07	7	241C>T, 3037C>T, 5209A>G, 14408C>T, 23403A>G, 24862A>G, 27923C>T
Tygerberg_08	6	241C>T, 3037C>T, 5209A>G, 14408C>T, 23403A>G, 24862A>G
Tygerberg_09	6	241C>T, 3037C>T, 5209A>G, 14408C>T, 23403A>G, 24862A>G
Tygerberg_10	8	241C>T, 3037C>T, 5209A>G, 11674C>T, 14408C>T, 23403A>G, 24862A>G, 25996G>C
Tygerberg_11	6	241C>T, 3037C>T, 5209A>G, 14408C>T, 23403A>G, 24862A>G
Tygerberg_12	7	241C>T, 3037C>T, 5209A>G, 13991A>G, 14408C>T, 23403A>G, 24862A>G
Tygerberg_13	5	241C>T, 5209A>G, 14408C>T, 23403A>G, 24862A>G
Tygerberg_14	7	241C>T, 626G>T, 3037C>T, 5209A>G, 14408C>T, 23403A>G, 24862A>G
Tygerberg_15	6	241C>T, 3037C>T, 5209A>G, 14408C>T, 23403A>G, 24862A>G
Tygerberg_16	6	241C>T, 3037C>T, 5209A>G, 14408C>T, 23403A>G, 24862A>G
Tygerberg_17	6	241C>T, 3037C>T, 5209A>G, 14408C>T, 23403A>G, 24862A>G
Tygerberg_18	7	241C>T, 3037C>T, 5209A>G, 9865T>G, 14408C>T, 23403A>G, 24862A>G
Tygerberg_19	7	241C>T, 3037C>T, 5209A>G, 13991A>G, 14408C>T, 23403A>G, 24862A>G
Tygerberg_20	6	241C>T, 3037C>T, 5209A>G, 11674C>T, 14408C>T, 23403A>G, 24862A>G, 25996G>C
Tygerberg_21	7	241C>T, 3037C>T, 5209A>G, 9865T>G, 14408C>T, 23403A>G, 24862A>G
Tygerberg_22	7	241C>T, 3037C>T, 5209A>G, 14408C>T, 19186C>T, 23403A>G, 24862A>G
Tygerberg_23	4	241C>T, 14408C>T, 23403A>G, 24862A>G
Tygerberg_24	6	241C>T, 3037C>T, 5209A>G, 14408C>T, 23403A>G, 24862A>G
Tygerberg_25	6	241C>T, 3037C>T, 5209A>G, 14408C>T, 23403A>G, 24862A>G
Tygerberg_26	6	241C>T, 3037C>T, 5209A>G, 14408C>T, 23403A>G, 24862A>G
Tygerberg_27	9	241C>T, 3037C>T, 5209A>G, 13748A>G, 14408C>T, 23403A>G, 24862A>G, 28373G>T, 29227G>T
Tygerberg_28	6	241C>T, 3037C>T, 5209A>G, 14408C>T, 23403A>G, 24862A>G
Tygerberg_29	7	241C>T, 3037C>T, 9223C>T, 13536C>T, 28881G>A, 28882G>A, 28883G>C
Tygerberg_30	12	241C>T, 3037C>T, 4002C>T, 10097G>A, 13536C>T, 14408C>T, 23403A>G, 23731C>T, 25169C>T, 28881G>A, 28882G>A, 28883G>C
Tygerberg_31	6	1515A>G, 9223C>T, 14805C>T, 17247T>C, 18568C>T, 26144G>T
Tygerberg_32	11	241C>T, 4002C>T, 10097G>A, 13536C>T, 14408C>T, 23403A>G, 23731C>T, 25169C>T, 28881G>A, 28882G>A, 28883G>C
Tygerberg_33	7	241C>T, 3037C>T, 14408C>T, 23403A>G, 28881G>A, 28882G>A, 28883G>C
Tygerberg_34	7	241C>T, 3037C>T, 14408C>T, 23403A>G, 24389A>C, 24390G>C, 28881G>A, 28882G>A, 28883G>C

Tygerberg_35	6	241C>T, 14408C>T, 23403A>G, 28881G>A, 28882G>A, 28883G>C
Tygerberg_36	7	241C>T, 3037C>T, 14408C>T, 23403A>G, 28881G>A, 28882G>A, 28883G>C
Tygerberg_37	6	241C>T, 5209A>G, 7798G>T, 14408C>T, 23403A>G, 24862A>G
Tygerberg_38	6	241C>T, 3037C>T, 5209A>G, 14408C>T, 23403A>G, 24862A>G
Tygerberg_41	8	241C>T, 6037C>T, 14408C>T, 23403A>G, 25792C>T, 28881G>A, 28882G>A, 28883G>C
Tygerberg_42	7	241C>T, 3037C>T, 14408C>T, 23403A>G, 28881G>A, 28882G>A, 28883G>C
Tygerberg_43	7	241C>T, 14408C>T, 23403A>G, 28300G>T, 28881G>A, 28882G>A, 28883G>C
Tygerberg_44	7	241C>T, 3037C>T, 8022T>G, 14408C>T, 23403A>G, 24389A>C, 24390G>C, 28881G>A, 28882G>A, 28883G>C
Tygerberg_45	7	241C>T, 3037C>T, 14408C>T, 23403A>G, 28881G>A, 28882G>A, 28883G>C
Tygerberg_46	9	241C>T, 3037C>T, 6555C>G, 6560T>G, 8022T>G, 14408C>T, 23403A>G, 24389A>C, 24390G>C, 28881G>A, 28882G>A, 28883G>C
Tygerberg_47	7	241C>T, 3037C>T, 14408C>T, 23403A>G, 28881G>A, 28882G>A, 28883G>C
Tygerberg_48	7	241C>T, 3037C>T, 8022T>G, 14408C>T,23403A>G, 24389A>C, 24390G>C, 28881G>A, 28882G>A, 28883G>C
Tygerberg_50	5	241C>T, 5209A>G, 14408C>T, 23403A>G, 24862A>G
